# Acute Pancreatitis Caused by Tirzepatide

**DOI:** 10.7759/cureus.76007

**Published:** 2024-12-19

**Authors:** Nur Mando, Erica Thomson, Matthew Fowler, Lillian Short, Nora Gillen

**Affiliations:** 1 Internal Medicine, Ascension Sacred Heart, University of Florida College of Medicine, Pensacola, USA; 2 General Surgery, Florida State University College of Medicine, Pensacola, USA

**Keywords:** drug-induced acute pancreatitis, glp-1 agonist, mounjaro, ozempic, tirzepatide

## Abstract

Glucagon-like peptide-1 (GLP-1) receptor agonists, including tirzepatide (Mounjaro), are widely used to manage type 2 diabetes mellitus (T2DM) and obesity. While gastrointestinal side effects are common, acute pancreatitis remains a rare but significant complication. Limited evidence exists on the risks associated with switching between GLP-1 agonists, emphasizing the need for clinical awareness.

We present a 59-year-old male with T2DM, hyperlipidemia, and hypertension, who was recently transitioned from semaglutide (Ozempic) to tirzepatide (Mounjaro). He presented with acute epigastric pain, nausea, and vomiting two days after initiating tirzepatide. Laboratory findings revealed elevated lipase levels (847 U/L), leukocytosis, and diagnostic imaging confirming acute pancreatitis with other causes ruled out. Supportive care improved symptoms initially, but the clinical course was complicated by fevers prompting repeat imaging, revealing worsening pancreatitis with colonic involvement and pleural effusion. The patient was treated with empiric antibiotics and supportive measures, resulting in resolution of symptoms. Tirzepatide was discontinued, with a follow-up arranged for glycemic management.

Acute pancreatitis is a rare but documented adverse effect of GLP-1 agonists, with limited cases reported in the literature. Switching between GLP-1 agonists may increase the risk of adverse effects, especially if appropriate dose titration protocols are not followed. This case highlights the recognition of acute pancreatitis as a potential adverse effect of GLP-1 agonists when initiating or transitioning GLP-1 therapies and following titration protocols to help avoid this complication.

GLP-1 agonists, including tirzepatide, offer significant therapeutic benefits for T2DM and obesity but carry risks of rare adverse effects like acute pancreatitis. Greater awareness, careful dose adjustments, and vigilant monitoring are essential to optimizing patient safety. Further research is needed to elucidate the safety profile of switching between GLP-1 agonists to guide clinical practice and improve patient outcomes.

## Introduction

Glucagon-like peptide-1 (GLP-1) agonists are a class of medications that have become especially popular and widely used to treat obesity and type 2 diabetes mellitus (T2DM). There are various GLP-1 agonists that are available in the United States and worldwide. Mounjaro, or tirzepitide, has become one of the most popular GLP-1 agonists on the market alongside Ozempic (semaglutide). Mounjaro, a dual glucose-dependent insulinotropic peptide (GIP) and GLP-1 receptor agonist, was approved by the US Drug Administration in May 2022 for patients with T2DM. This drug works by stimulating insulin release and promoting the secretion of the natural incretin hormones. Incretin hormones GIP and GLP-1 work by increasing insulin release, thereby decreasing serum blood glucose levels. GLP-1 also inhibits glucagon secretion, which is also helpful in treating diabetes because glucagon is the hormone that promotes the release of blood glucose in the body in a fasting state. Mounjaro can also suppress appetite as it delays gastric emptying [1]. For this reason, Mounjaro and other medications in the same class are used off-label for weight loss in overweight and obese patients [2]. The side effect profile of Mounjaro is well documented and mainly includes gastrointestinal symptoms such as dyspepsia, emesis, nausea, diarrhea, and constipation. Acute pancreatitis is considered a rare side effect.

## Case presentation

**Figure 1:**
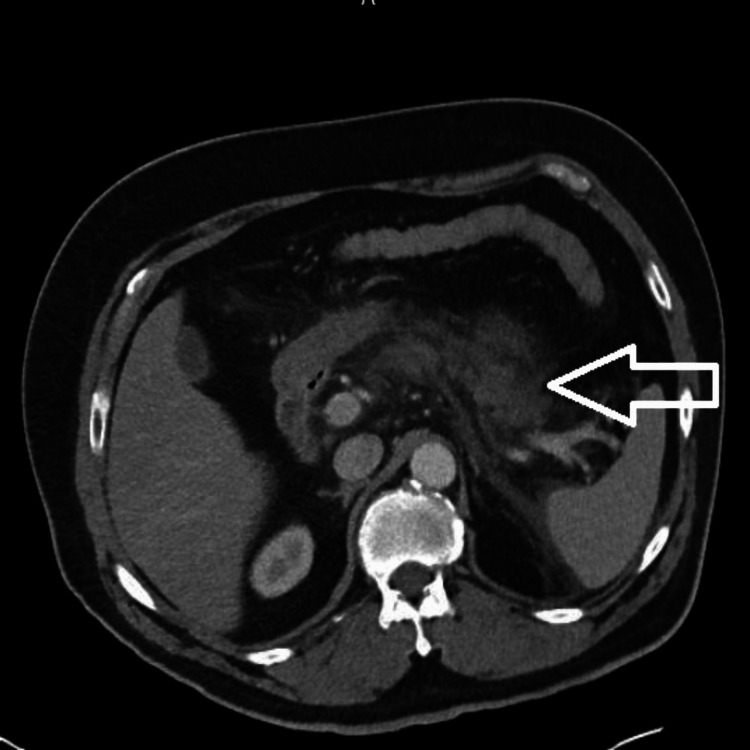
CT abdomen/pelvis performed at ED on arrival revealing extensive and diffuse peripancreatic stranding with surrounding fluid, which extends into the splenic hilum and along the undersurface of the spleen, as well as throughout the left pararenal space The arrow points towards acute pancreatitis. ED: Emergency department

A 59-year-old Caucasian male with T2DM, hyperlipidemia, and hypertension presented to the emergency department (ED) with epigastric pain. The patient reported experiencing generalized fullness in his abdomen two days prior to arrival and initially thought he was just bloated/constipated. The next day, his symptoms progressed to associating nausea and several episodes of non-bloody emesis. Later that same day, he subsequently developed severe upper abdominal pain from the epigastric to the periumbilical regions. The patient denied any previous history of pancreatitis, and he had never experienced similar symptoms before. He denied any alcohol or illicit drug use. However, he reported that he had been taking Ozempic 1mg injections weekly and had lost around 20 pounds in the previous six months. The patient reported that his provider switched him from Ozempic to Mounjaro just the week before arrival; he was switched from 1mg Ozempic injections weekly to 7.5mg Mounjaro injections. He had just started his new dose of Mounjaro the day his symptoms started. On arrival, the patient met systemic inflammatory responses (SIRs) criteria with leukocytosis (white blood count (WBC) 13) and tachycardia (heart rate (HR)>90 bpm). Other lab work significant for hemoglobin (Hgb) 14.0, Hct of 39.6, blood urea nitrogen (BUN) 14, calcium (Ca) 9.2, albumin 3.7, and a lipase of 847. Troponin was within normal limits. Urinalysis revealed only glucosuria. Bilirubin and liver enzymes were within normal limits. Influenza A and B, Covid-19, and respiratory syncytial virus (RSV) were negative. A CT of the abdomen/pelvis was performed on arrival, which revealed extensive and diffuse peripancreatic stranding with surrounding fluid, which extended into the splenic hilum and along the undersurface of the spleen, as well as throughout the left pararenal space (Figure 1). The right upper quadrant ultrasound was negative for cholelithiasis or cholecystitis. A lipid panel was obtained, and no evidence of hypertriglyceridemia was found. His glycated Hgb (HbA1c) at the time of hospitalization was 6.1%.

**Figure 2:**
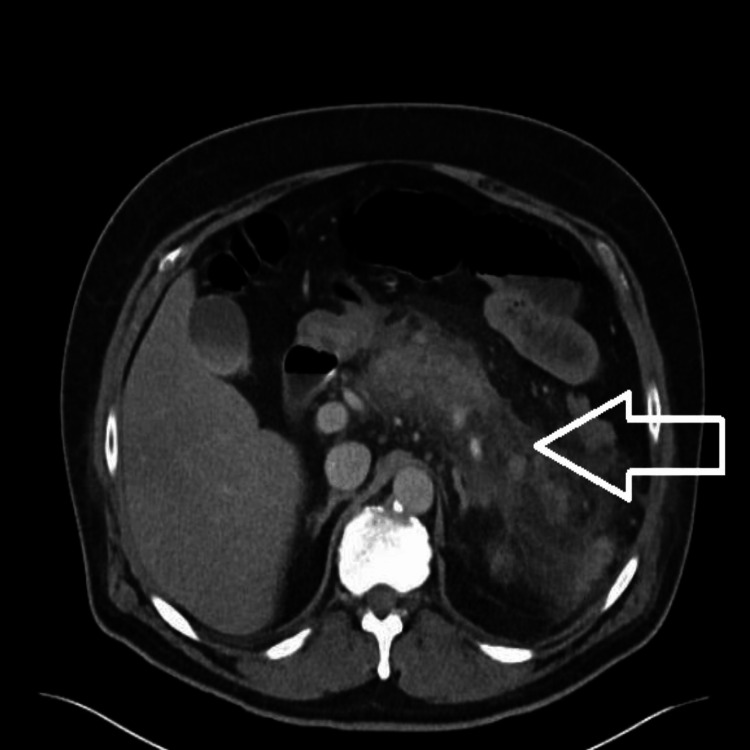
Repeat imaging showing worsening in pancreatitis The arrow points towards worsening acute pancreatitis.

The patient was admitted to the hospital for evaluation and treatment of acute pancreatitis. The patient was monitored in the hospital and given supportive care measures, including pain control, and symptoms slowly improved. Gastroenterology was consulted to assist in the patient's care. After an extensive medication review with the patient and after a comprehensive medical history was acquired, his GLP-1 agonist was suspected to be the cause of his pancreatitis. Unfortunately, the patient experienced persistent fevers during his hospitalization, ranging from 38-39°C. This prompted a repeat CT scan on his fifth day of hospitalization that showed worsening pancreatitis with involvement of his left colonic wall, evidence of ileus, and bibasilar lung atelectasis with a left-sided pleural effusion (Figure 2). He was initiated on empiric antibiotic therapy and continued on IV fluids as recommended by gastroenterology. The patient improved and his diet was progressed and his ileus resolved. He was discharged with close follow-up planned with gastroenterology and instructed to discontinue Mounjaro.

## Discussion

The mechanism of Mounjaro helps to mitigate blood glucose levels in diabetics because it increases serum insulin levels released by the pancreas and decreases the hormone responsible for the release of blood glucose, glucagon [3]. A meta-analysis determined that all GLP-1 receptor agonists lead to significant weight reduction. However, tripeptide was associated with better efficacy while maintaining a similar safety profile [4]. Treating T2DM with tirzepatide has been associated with improved quality of life by patient-reported data because of the decreased weight loss and lower HbA1c [5]. This particular trial used the SURPASS-1 to-5 trials used in the original studies that determined the efficacy of the drug and led to the United States Food and Drug Administration (FDA) approval. They followed up with the patients to determine their quality of life and found that their quality of life was improved compared to those who were taking metformin. Serious side effects of the drug include hypoglycemia, acute pancreatitis, cholelithiasis, and cholecystitis, with less serious side effects such as nausea, vomiting, diarrhea, constipation, and dyspepsia.

Gastrointestinal (GI) side effects are the number one reported reason for non-compliance. Some research shows that nausea and vomiting can be prevented with vitamin B12 and corrin ring-containing compounds [6]. Interestingly, a systematic review and meta-analysis on the adverse effects of Mounjaro showed that the incidence of acute pancreatitis, cholelithiasis, and cholecystitis was similar regardless of drug dosage. In contrast, the more common side effects of nausea and vomiting were dose-dependent [7]. There are so few cases reported in the literature of pancreatitis caused by tirzepatide, which may decrease clinician awareness about this side effect and skew research that relies on available research for analysis. Pancreatitis, for example, as a side effect of the drug was deemed potentially safe by a meta-analysis and systematic review on the tirzepatide's side effect profile, although the pharmaceutical companies document it as a known side effect [8]. If clinicians do not ask if the patient is taking a GLP-1 agonist they may never report this etiology of pancreatitis. Likewise, they may not ask pancreatitis patients about the use of the drug or query the patient about a history of pancreatitis before prescribing the drug. Perhaps the rarity of cases and the limited data skewed the analysis of the data acquired in the literature review. Knowing about this side effect is important because acute pancreatitis can be a devastating disease. 

More than 235,000 Americans are admitted to hospitals with a diagnosis of acute pancreatitis yearly. Pancreatitis can present with epigastric pain, nausea, fever, and chills, with lab values showing elevated lipase levels and leukocytosis. The diagnosis of pancreatitis requires two of the three criteria to be met, including epigastric abdominal pain, an elevated serum lipase three times above the normal limit, and radiological signs of pancreatitis [9]. Laboratory values can indicate volume depletion with hemoconcentration and leukocytosis. Volume status can be evaluated by physical exam findings and supplemented by labs such as creatinine, lactate, hematocrit, and BUN. The severity of acute pancreatitis can vary tremendously from an uneventful hospital course to sepsis, multi-organ failure, and death. Treatment requires supportive care, including fluid resuscitation, pain control, organ function assessments, and the appropriate interventional therapies such as endoscopic retrograde cholangiopancreatography (ERCP), sphincterotomy, and cholecystectomy if the disease severity progresses. Certain severity scores, such as the Ranson score, the APACHE-II score, or the Atlanta criteria, can be used to classify the disease, help indicate the outcome of this pancreatitis, and guide early management [10]. It is also essential to find the cause of pancreatitis, which will guide treatment. Alcohol use and gallstone pancreatitis are the most common causes of pancreatitis in the United States. However, there are other etiologies of pancreatitis, such as drug-induced pancreatitis as in this case [11]. 

While it is imperative to note the risk of acute pancreatitis with initiation of a GLP-1 agonist alone, it is crucial to understand the potential risk of GI symptoms and potential for acute pancreatitis when switching between different GLP-1 agonist medications. Patients may sometimes switch from different GLP-1 receptor agonists for various reasons, whether due to cost, efficacy, or side effects. When starting subcutaneous treatment, it is well documented and recommended to start with the lowest dose and titrate upwards. If a patient is switching GLP-1 agonists due to GI symptoms, they should start the new GLP-1 agonist at the lowest dose, regardless of the dose the patient received from the previous medication [12]. If switching for another reason, they should start the new GLP-1 agonist at the same or lower dose. 

## Conclusions

Acute pancreatitis and adverse effects of switching GLP-1 agonists are not well documented and it is important to raise awareness of these potential complications. This gap in literature and lack of awareness highlights a crucial area of opportunity for discussions and shared decision-making regarding the management for these medications. Furthermore, encouraging discussions among healthcare providers and patients is essential to ensure the timely identification of adverse events to promote a proactive and informed approach to GLP-1 agonists. As the prevalence of T2DM and obesity continues to rise globally, ongoing research into the long-term safety profile of these medications, including the risks of pancreatitis, is vital. Only through continued investigation can we ensure that patients receive the safest, most effective treatments while minimizing the risk of severe adverse events.
